# Adenomatous Polyposis Coli Regulates Axon Arborization and Cytoskeleton Organization via Its N-Terminus

**DOI:** 10.1371/journal.pone.0024335

**Published:** 2011-09-06

**Authors:** Youjun Chen, Xu Tian, Woo-Yang Kim, William D. Snider

**Affiliations:** 1 Department of Cell and Molecular Physiology and Neuroscience Center, School of Medicine, University of North Carolina at Chapel Hill, Chapel Hill, North Carolina, United States of America; 2 Hefei National Laboratory for Physical Sciences at Microscale and School of Life Sciences, University of Science and Technology of China, Hefei, Anhui Province, People's Republic of China; 3 Department of Developmental Neuroscience, Munroe-Meyer Institute, University of Nebraska Medical Center, Omaha, Nebraska, United States of America; Virginia Commonwealth University Medical Center, United States of America

## Abstract

Conditional deletion of *APC* leads to marked disruption of cortical development and to excessive axonal branching of cortical neurons. However, little is known about the cell biological basis of this neuronal morphological regulation. Here we show that APC deficient cortical neuronal growth cones exhibit marked disruption of both microtubule and actin cytoskeleton. Functional analysis of the different APC domains revealed that axonal branches do not result from stabilized β-catenin, and that the C-terminus of APC containing microtubule regulatory domains only partially rescues the branching phenotype. Surprisingly, the N-terminus of APC containing the oligomerization domain and the armadillo repeats completely rescues the branching and cytoskeletal abnormalities. Our data indicate that APC is required for appropriate axon morphological development and that the N-terminus of APC is important for regulation of the neuronal cytoskeleton.

## Introduction

APC is an important tumor suppressor that regulates β-catenin levels in the wnt signaling pathway [1]. In addition to the β-catenin binding region, APC contains multiple functional domains, each of which binds to proteins that regulate key cellular processes. APC is thought to have important functions related to cell polarity, microtubule stability and cell migration based on *in vitro* studies [2].

Previously we and another group showed that conditional loss of APC in neural progenitor cells severely disrupted the structure of the developing cerebral cortex as well as axon projections *in vivo* [3], [4]. Further, we found that dissociated APC-deficient cortical neurons exhibit exuberant axon branching *in vitro* [4]. Other recent studies have demonstrated that APC is involved in axon guidance of retinal ganglion cells by its differential distribution at the growth cone [5] and that knock down of APC in dorsal root ganglion (DRG) neurons leads to microtubule looping in the growth cone [6]. However the cell biological basis of this APC growth cone regulation remains unclear and the APC domains required to regulate neuronal morphology have not been specified.

Extensive domain analysis of the APC protein has been carried out in non-neuronal cells [7]. In addition to the β-catenin binding domain, the key structural motifs include the oligomerization domain, the armadillo repeats that bind to the IQ-motif-containing GTPase-activating protein (IQGAP), the APC-stimulated guanine nucleotide exchange factors (ASEFs) and kinesin associated protein 3 (KAP3) [8], [9], [10], the carboxyl-terminus that binds to microtubules and microtubule plus end-binding protein 1 (EB1) [11] and the mammalian homolog of Discs large (DLG1) binding region that is thought to be important for cell cycle and cell polarity regulation [12], [13]. It is plausible that all domains of APC are required to mediate its morphological regulation of neurons. On the other hand, functions mediated by a specific domain might be most important.

Prior investigations in neurons including our previous study have been directed at functions of the entire protein with shRNA knockdown or dominant inhibitory approaches [14], [15]. We took advantage of the striking morphological abnormalities that we have observed in neurons that lack APC altogether. We introduced the various APC-domains into APC deficient neurons and assessed their ability to rescue morphology.

We have found that both actin and microtubule organization are severely disrupted in the growth cones of APC deleted neurons well prior to axon branch formation. Because of the microtubule abnormalities, we hypothesized that expression of the C-terminus of APC might be sufficient to suppress branch formation in APC deleted neurons. However, surprisingly, neither the microtubule binding domain, nor the EB1 binding domain, nor both together fully rescued the phenotype. Instead, expression of the amino terminus, containing the oligomerization domain and the armadillo repeats, mediated complete rescue. We conclude that N-terminal region of APC has important functions in the regulation of neuronal cytoskeleton.

## Materials and Methods

### Mice

All procedures were carried out according to an animal protocol (protocol number: 11-029.0) approved by the Institutional Animal Care and Use Committee (IACUC) at University of North Carolina-Chapel Hill. APC^lox/lox^Nestin-Cre^+^ embryos [4] were generated by mating mice harboring an APC floxed allele [16] with Nestin-Cre mice [17]. Since no differences were observed between heterozygote embryos (APC^lox/+^ Nestin-Cre^+^) and wild type littermates (APC^lox/lox^ Nestin-Cre^−^ or APClox/+ Nestin cre-), both heterozygous and wild-type littermates were used as controls. Genotypes were determined by PCR using primers specific for the APC floxed allele, the APC wild type allele, and cre.

### DNA constructs

DNA constructs were amplified and purified with by EndoFree Plasmid Maxi kits (QIAGEN Sciences).

The N-terminal truncation mutant APC-N, and the C-terminal truncation mutants APC-C, APC-C1, and APC-C2 expression vectors were generated as described previously [15]. APC-N1 and APC-N2 expression vectors were kindly provided by Dr Inke Nathke [18]. The stabilized β-catenin expression vector was kindly provided by Dr Fengquan Zhou. The inhibitor of β-catenin and TCF-4 (ICAT) expression vectors were kindly provided by Dr Anjen Chenn [19]. To obtain the APC-N1-C2 expression vector, we first subcloned the APC-N1 cDNA fragment into Hind III and Sal I sites of pEGFP-C1 vector. The APC-C2 cDNA fragment was then subcloned into the (using Sal I and BamH I sites) of pEGFP-C1-APC-N1 construct.

### Primary neuronal cultures

Cerebral cortices dissected from mouse E14-E16 embryos were treated with trypsin for 5 min and washed with minimum essential medium (MEM) containing 10% fetal bovine serum before dissociation. Neurons were plated onto glass coverslips (for morphology analysis) or dishes (for western blot analysis) coated with poly-D-lysine and laminin and maintained in neurobasal A medium containing B27 supplement, L-Glutamine and Penicillin/Streptomycin. Neurons were cultured for 1 to 7 days *in vitro* (DIV) as indicated and were fixed or harvested for further analysis. Cell densities were around 300 neurons per cm^2^ for axon branching analysis and around 10^5^ neurons per cm^2^ for western blot analysis.

### Neuronal transfection

DNA constructs were introduced into cortical neurons using an electroporation technique from Lonza (Amaxa Mouse Neurons Nucleofector Kit). The procedure was performed according to the protocol described in the kit. Briefly, dissociated mouse cortical neurons were suspended in 100 µl of Amaxa electroporation buffer mixed with 10 ug of DNA. After electroporation, neurons were immediately plated on laminin/poly-D-lysine coated coverslips. Neurons were cultured for 1 to 4 days as indicated before fixation and immunocytochemistry for further analysis.

### Westernblot

Cells were lysed in RIPA buffer (1% NP40, 0.25% sodium deoxycholate, 1 mM EGTA, 150 mM NaCl, 50 mM Tris, pH 7.5) supplemented with protease inhibitor cocktail (Sigma, St. Louis, MO). Cell protein was determined by Bio-Rad protein assay (Bio-Rad Laboratories, Inc., Hercules, CA) and separated by SDS-PAGE gels. Blots were incubated with primary antibodies APC (C-20, rabbit polyclonal, 1∶50, Sana Cruz biotechnology, Santa Cruz, CA), β-catenin (1∶5000, rabbit polyclonal, Cell Signaling Technology, Beverly, MA), or α-tubulin (mouse monoclonal, 1∶10000, Sigma, St. Louis, MO) at 4 degrees overnight. After three times of washing with PBS, blots were incubated in horseradish peroxidase-conjugated secondary anti-mouse (1∶10000, Dako, Carpinteria, CA) or anti-rabbit (1∶2000, Cell Signaling Technology, Beverly, MA) antibodies and were developed using enhanced chemiluminescence (GE Healthcare, Waukesha, WI).

### Immunocytochemistry and Antibodies

Neurons were fixed with 4% parafomaldehyde in 0.1 M sodium phosphate buffer (pH 7.2) for 20 minutes, and permeabilized and blocked with 2% BSA in PBS containing 0.1% triton X-100 and NaN3. Then neurons were incubated with primary antibody at 4°C overnight, followed by secondary antibody incubation for 1 hour.

Primary antibodies used were APC (C-20, rabbit polyclonal, 1∶50, Sana Cruz biotechnology, Santa Cruz, CA), α-tubulin (mouse monoclonal, 1∶1000, Sigma, St. Louis, MO), Tau1 (mouse monoclonal, 1∶500, Millipore, Billerica, MA), MAP2 (rabbit polyclonal, 1∶500, Millipore, Billerica, MA), GFP (rabbit polyclonal, 1∶1000, Invitrogen, Eugene, OR). All fluorescence-labeled secondary antibodies (1∶1000) were from Molecular Probes (Eugene, OR). Fluorescence-labeled phalloidin (1∶40) was from Molecular Probes (Eugene, OR).

### Acquisition of images

Images of cortical neurons stained with indicated antibodies were acquired either by Metamorph under a Nikon SMZ1500 wide field fluorescence microscope (for branching analysis) or LSM (Laser Scanning Microscopy) software under the Zeiss 510 confocal microscope (for cytoskeleton analysis). When neurons were visualized by the wide field microscope, around 50 consecutive fields for each experimental condition were imaged to avoid selection bias. When neurons were visualized by confocal microscope, around 30 consecutive fields for each experimental condition were imaged. For domain analysis, since neuronal transfection efficiency is very low, all GFP positive neurons were imaged when there were less than 50 GFP positive neurons.

### Analysis of axon branching

Images were analyzed with MetaMorph software (version 7.6.2.0, Molecular Devices, Inc. Sunnyvale, CA). Tau1-positive neurites were counted as axons and MAP2-positive neurites were counted as dendrites. Only branches longer than 20 µm from the parent axon were included in the analysis. Primary branches are branches initiating from the major axons and secondary branches are branches initiating from primary branches. Total branches are the sum of primary branches, secondary branches and higher degree branches from all the axons of each neuron. The difference between control and APC cKO neurons was assessed by student t-test.

### Time lapse imaging

Neurons were first cultured on poly-D-lysine and laminin coated glass bottom dishes for 1DIV or 3DIV as indicated. During imaging, culture dishes were set on microscope (Nikon SMZ1500) stage with a temperature and CO2 monitoring chamber (LiveCell system by Neue Biosciences).

## Results

### APC elimination leads to excessive axon branching

**Figure 1:**
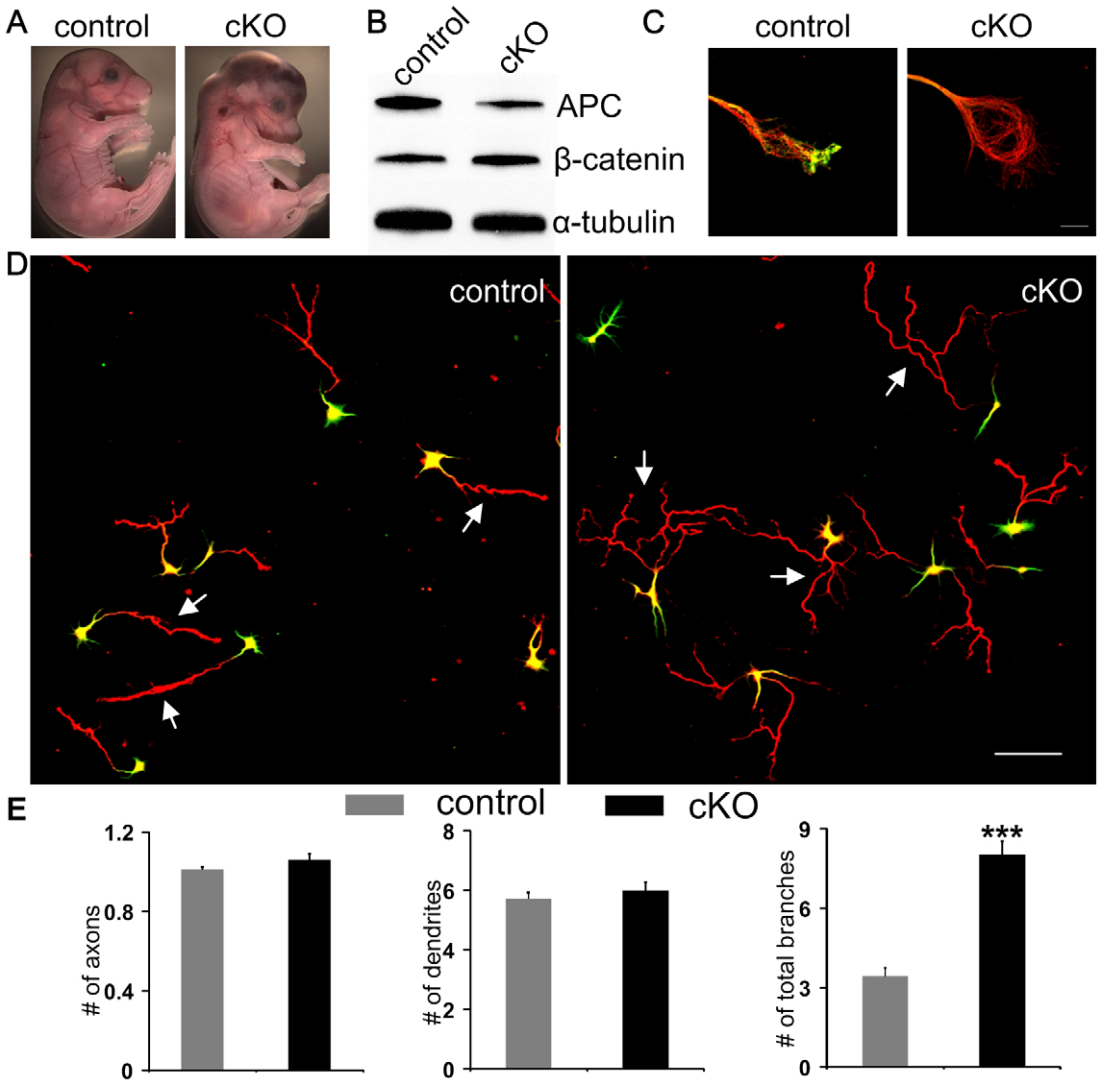
Deletion of APC induces excessive axon branching. (A) Control (left) and APC-cKO mouse embryos (right) at E16.5. (B) Western blot of dissociated control and APC cKO cortical cultures. Residual APC is likely due to non-neural cells in the culture derived from cells that did not undergo recombination. β-catenin protein levels were increased as expected. α-tubulin was used as a loading control. (C) Immunostaining showing APC accumulation at the growth cone tip in control axons (left) and absence of APC in APC-deficient axons (right). (D) Control and APC deficient cortical neurons cultured for four days and double labeled with antibodies against axon marker Tau1 (red) and dendritic marker MAP2 (green). Note that most APC-deleted neurons have highly branched axons (arrows). (E) Average numbers of axons, dendrites, and total axonal branches per neuron. Values are means ± SEM. ***: P<0.001 by student t test. More than 5 independent experiments were performed. Scale bars: C 5 µm, D 100 µm.

APC conditionally mutant embryos were easily identified by the abnormally shaped head from embryonic day 14 (E14) till late embryonic stages (Fig. 1A). Neuronal cultures were prepared from the developing cerebral cortex at embryonic day E14–E16 as described in our previous paper [4]. We first confirmed reduction of APC protein in cortical neurons cultured *in vitro*. Western blot analysis showed major reduction in APC protein levels (Fig. 1B). The residual protein was a result of cells not expressing cre recombinase in the culture, such as fibroblasts. As expected, β-catenin protein levels were increased. To further confirm the elimination of APC in APC-deficient neurons, we immunostained neurons with an APC antibody. Available APC antibodies appeared to be not entirely specific for immunocytochemistry [20]. However, immunostaining using the APC C-20 antibody from Santa Cruz verified absence of APC accumulation at axon tips of APC deficient cortical neurons (Fig. 1C). Quantification of fluorescence intensity at the axon tips verified that APC was absent in cortical neurons cultured from APC^lox/lox^Nestin-Cre^+^mice (Fig. S1).

To determine whether loss of endogenous APC had any effect on axon/dendritic specification of cortical neurons, we cultured cortical neurons from both APC cKO embryos and control littermates at E14.5 to E16.5 for four days *in vitro* (4DIV). By that stage, most control neurons are fully polarized, typically with one axon and several dendrites (Fig. 1D, left panel). Interestingly, we found that neurons from APC cKO embryos also had one axon and several dendrites (Fig. 1D, right panel). Quantification showed that the average numbers of axons and dendrites of APC cKO neurons were not significantly different from those of control neurons (Fig. 1E). On the other hand, axons from APC deficient neurons were highly branched compared to controls as we had demonstrated previously (Fig. 1D, arrows; Fig. 1E). While control neurons usually have few short branches extended from the major axon shaft, there are many long branches from APC deficient axons. These results demonstrate that APC is not required for axon/dendrite specification but is important for axon morphogenesis.

### Growth cones split in APC-deficient axons

**Figure 2:**
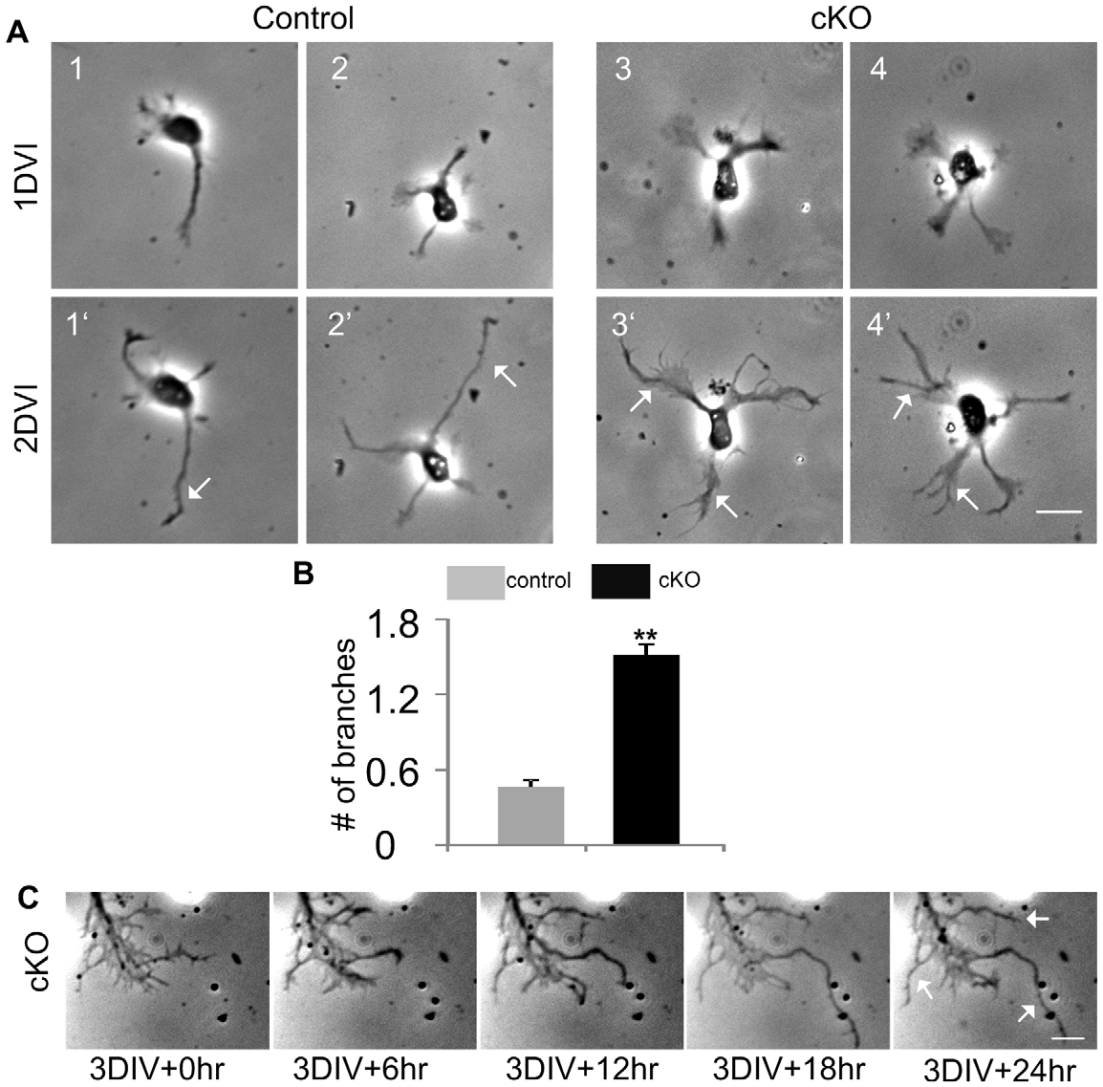
APC deletion induces growth cone splitting. (A) Representative images of the same neurons taken at 1DIV (top panel) and 2DIV (bottom panel) from control and APC deleted embryos. Note that control growth cones normally remain intact (1′ and 2′, arrows) at 2DIV, while APC deficient growth cones split into multiple branches (3′ and 4′, arrows). (B) The average number of branches that emanated from control and APC deficient growth cones at 2DIV. (C) Phase-contrast images of an APC cKO axon observed over 24 hr in time lapse. Cortical neurons from around E15 embryos were cultured for 3 days *in vitro* before imaging. **: P<0.005 by two-tailed student t-test. Data are presented as mean ± SEM. Scale bars: 20 µm.

To investigate how supernumerary axon branches extend from APC-deficient axons, we performed time lapse imaging to monitor branch sprouting *in vitro*. We imaged neurons with relatively intact growth cones at 1DIV (Fig. 2A, top panels) and the same neurons again at 2DIV (Fig 2A, bottom panels). A striking phenomenon we observed from APC deficient neurons is that growth cones frequently split, producing multiple branches simultaneously. At 2DIV, about 70% of APC deficient growth cones split into multiple branches while less than 30% of control growth cones formed branches. APC deficient growth cones split into almost three times as many branches as controls on average (Fig. 2B). This phenomenon was also observed in fully differentiated axons, imaged from 3DIV. An example with 4 branches emanating from a growth cone is shown in Fig. 2C (arrows). Although branches sometimes arose from control growth cones, we rarely observed control growth cones splitting into multiple branches. These results indicate that APC is important for maintenance of growth cone morphology.

### APC is important for neuronal cytoskeletal organization

APC associates with microtubules and multiple studies have demonstrated that APC is important for microtubule polymerization and is involved in directing microtubule plus tips to the leading edge of migrating cells [21], [22], [23]. In neurons, APC is accumulated at the axon tips, particularly at microtubule plus ends and plays an essential role in axon growth and growth cone steering [5], [14], [15], [24]. Previously we observed debundling of microtubules at the branch points of APC null axons [4], we hypothesized that microtubules were debundled and disorganized before branch formation. To test this hypothesis, we first examined microtubule and actin organization prior to growth cone splitting into branches at 1DIV.

**Figure 3:**
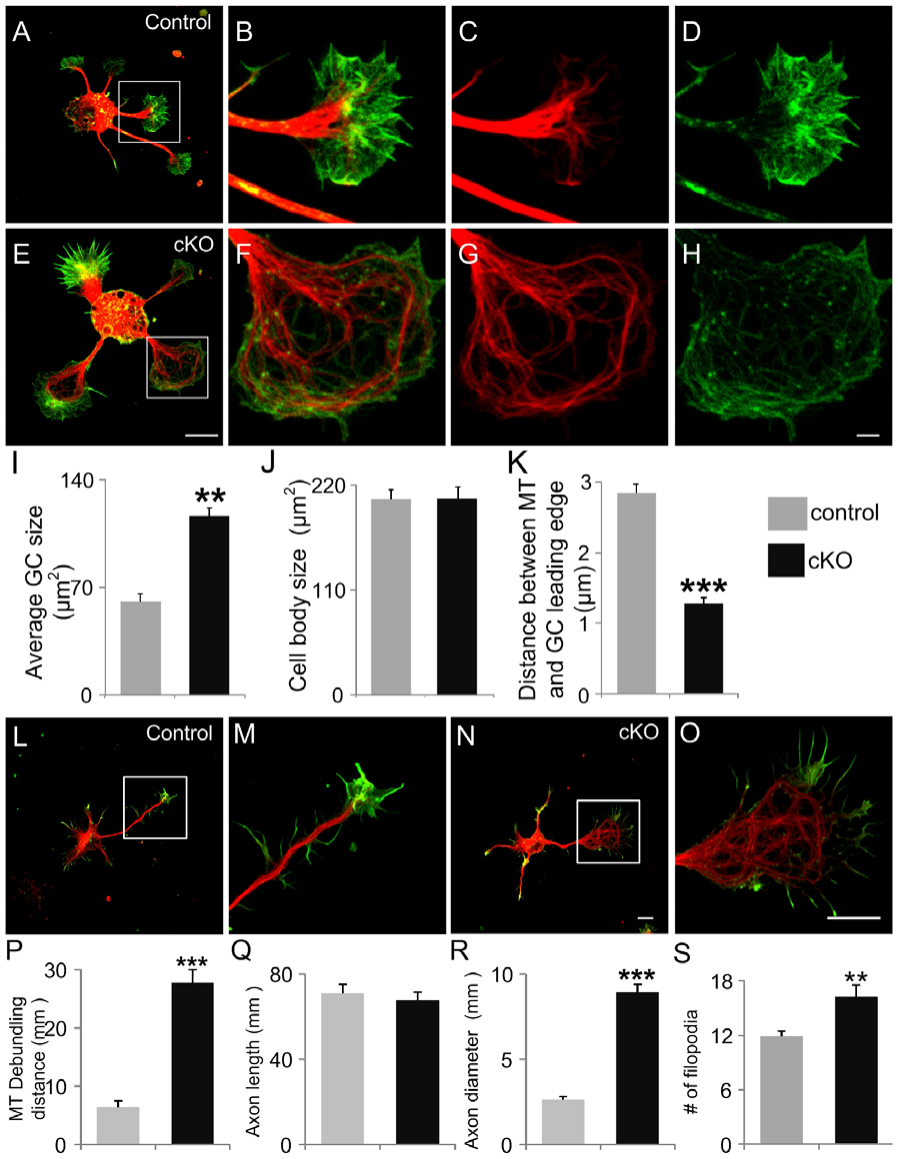
APC deletion induces cytoskeleton reorganization at axonal growth cones. (A and E) Control (A) and APC cKO (E) cortical neurons fixed at 1DIV and double stained with phalloidin (green) and anti-α-tubulin antibody (red). (B–D and F–H) Higher magnification images of control (B–D) and APC cKO (F–H) growth cones from the inserts shown in A and E. Note that microtubules gradually splay apart and extend towards the central domain of the growth cone in control neurons (C); in contrast, microtubules are highly debundled and disoriented in APC deficient growth cones. Disruption of F-actin allows microtubules to reach the growth cone leading edge (G). (I) Growth cone sizes of control and APC cKO neurons. (J) Cell body sizes of control and APC cKO neurons. (K) Distances between microtubule tips and the growth cone leading edge. Note microtubule tips frequently reach the leading edge in APC cKO growth cones. (L and N) Control (L) and APC cKO (M) cortical neurons fixed at 2DIV and double labeled with phalloidin (green) and anti-α-tubulin antibody (red). (M and O) High-magnification images of control and APC cKO growth cones from the inserts shown in L and N. (P) Distance of the debundled microtubules measured from the axon distal end to the point at axon shaft where microtubules are tightly bundled. Note that microtubules are debundled for a long distance at the distal part of APC cKO axons while microtubule debundling is restricted at the tip of control axons. (Q) Axon length. (R) Axon diameter at the thickest point. (S) Filopodia number at the growth cone of control and APC cKO neurons. n>60 neurons for both control and APC cKO conditions. **: P<0.005 and ***: P<0.001 by two-tailed student t-test. Data are presented as mean ± SEM. Scale bars: (A and E) 10 µm; (B–D and F–H) 2 µm; (L–O) 10 µm.

In control neurons, microtubules were tightly bundled along the shaft and became gradually de-bundled in the central domain of the growth cone. Lamellipodia and filopodia were easily distinguished in control growth cones. A few microtubules extended to the transition domain and peripheral domain where they were usually aligned alongside filopodia (Fig. 3A–D). Strikingly, microtubules in the growth cones of APC null neurons were highly disrupted. They were dramatically splayed apart and disoriented. Instead of extending toward the growth cone leading edge, microtubules were arranged irregularly without any clear direction (Fig. 3E–G). This abnormal microtubule organization was observed in the growth cones of most of the neurites from APC-deficient neurons. Microtubule debundling resulted in a two-fold increase in average growth cone size of APC-deleted compared to control neurons (Fig. 3I).

In growth cones with abnormal microtubule structure, actin filaments were also disorganized. Instead of lamellipodia and filopodia in the peripheral domain of the growth cone normally seen in wild type neurons, actin filaments were misoriented, and a distinct lamellopodial domain was lacking in many APC deficient growth cones (Fig. 3H). Microtubule extension to the leading edge is normally inhibited by an actin barrier in the transition and peripheral domains in control growth cones [25]. In many APC deficient neurons, it seems that the actin barrier did not function properly. Microtubules were no longer restricted in the central domain and in fact, many microtubules were observed in the leading edge of growth cone (Fig. 3G). Quantification of these observations showed that the average distance between microtubules and the leading edge was greatly shortened in APC-deleted neurons (Fig. 3K). These findings demonstrate that APC is important for directional extension, bundling and proper distribution of microtubules in the growth cone. A plausible explanation for our results would be that F-actin disruption in APC deficient growth cones prevents proper regulation of microtubule extension. Indeed, the interaction between microtubules and F-actin is known to be important during axon guidance and growth cone steering [26].

In order to assess APC regulation of microtubules in extending axons, we selected neurons with a presumptive axon and few branches at 2DIV. In control neurons, microtubule debundling is restricted to the central domain of the growth cone within a short distance from the leading edge (Fig. 3L–M). In contrast, microtubule debundling occurs throughout the distal part of APC-deficient axon (Fig. 3N–P). As a result, APC-deficient axons were markedly thicker than controls (Fig. 3R). Further, microtubule organization in the growth cone was grossly disrupted. Microtubule bundles were curled and lacking apparent orientation. Growth cone lamellopdia were also disrupted. Surprisingly however, filopodia could form and a striking finding is that APC deletion substantially increased filopodia formation at the growth cone (Fig. 3S). Taken together, these data demonstrate that APC is important for microtubule and actin organization at the growth cone.

### The N-terminus of APC rescues neuronal morphology

**Figure 4:**
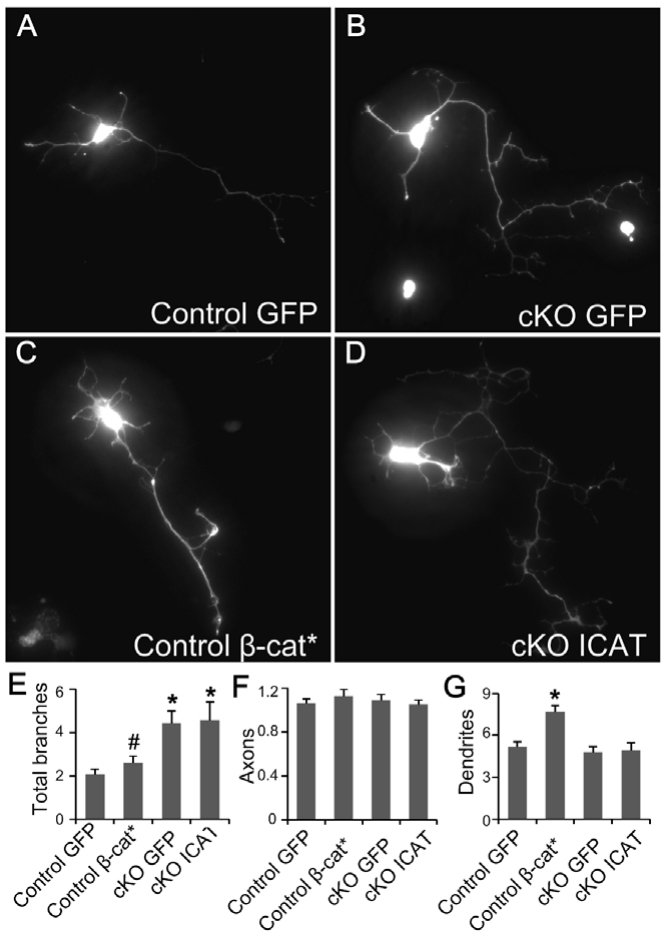
Stabilized β-catenin does not induce branch formation. (A–D) Control cortical neurons expressing GFP (A) or stabilizedβ-catenin (β-cat*, C), and APC cKO cortical neurons expressing GFP (B) or ICAT (D). Cortical neurons were cultured for 4 days in vitro after dissociation and transfection. (E) The number of total branches per neuron. (F) The average axon number per neuron. (G) The average dendrite number per neuron. *: p<0.005, when compared with GFP expressing control neurons. #: P<0.005, when compared with GFP expressing APC deficient neurons. n>120 neurons for each conditions from three independent experiments. Data are presented as mean ± SEM.

One major function of APC is its ability to control cellular β-catenin levels [27](Bienz & Clevers, 2000). To address the possibility that increased β-catenin could affect axon branching morphology, we expressed a stabilized form of β-catenin (β-cat*) in control cortical neurons. β-cat* is a mutated form of β-catenin with four serine/threonine residues (Ser^33^, Ser^37^, Thr^41^, and Ser^45^) changed to alanine and thereby resistant to proteasome mediated degradation [28], [29]. We did not observe a significant difference of the numbers of axons and axonal branches between control neurons expressing GFP alone and those expressing β-cat* (Fig. 4A, C and E). We did notice that the number of dendrites was increased in β-cat* expressing neurons (Fig. 4G), similar to what has been reported previously [30]. We then expressed the inhibitor of β-catenin and TCF-4 protein (ICAT) [31] in APC deficient neurons. The morphology of neurons expressing ICAT was similar to APC deficient neurons expressing GFP alone and the number of axonal branches was not significantly different between GFP and ICAT expressing APC deficient neurons (Fig. 4B, D and E). Our results suggest that increased axonal branching in APC deficient neurons is independent of stabilized β-catenin.

**Figure 5:**
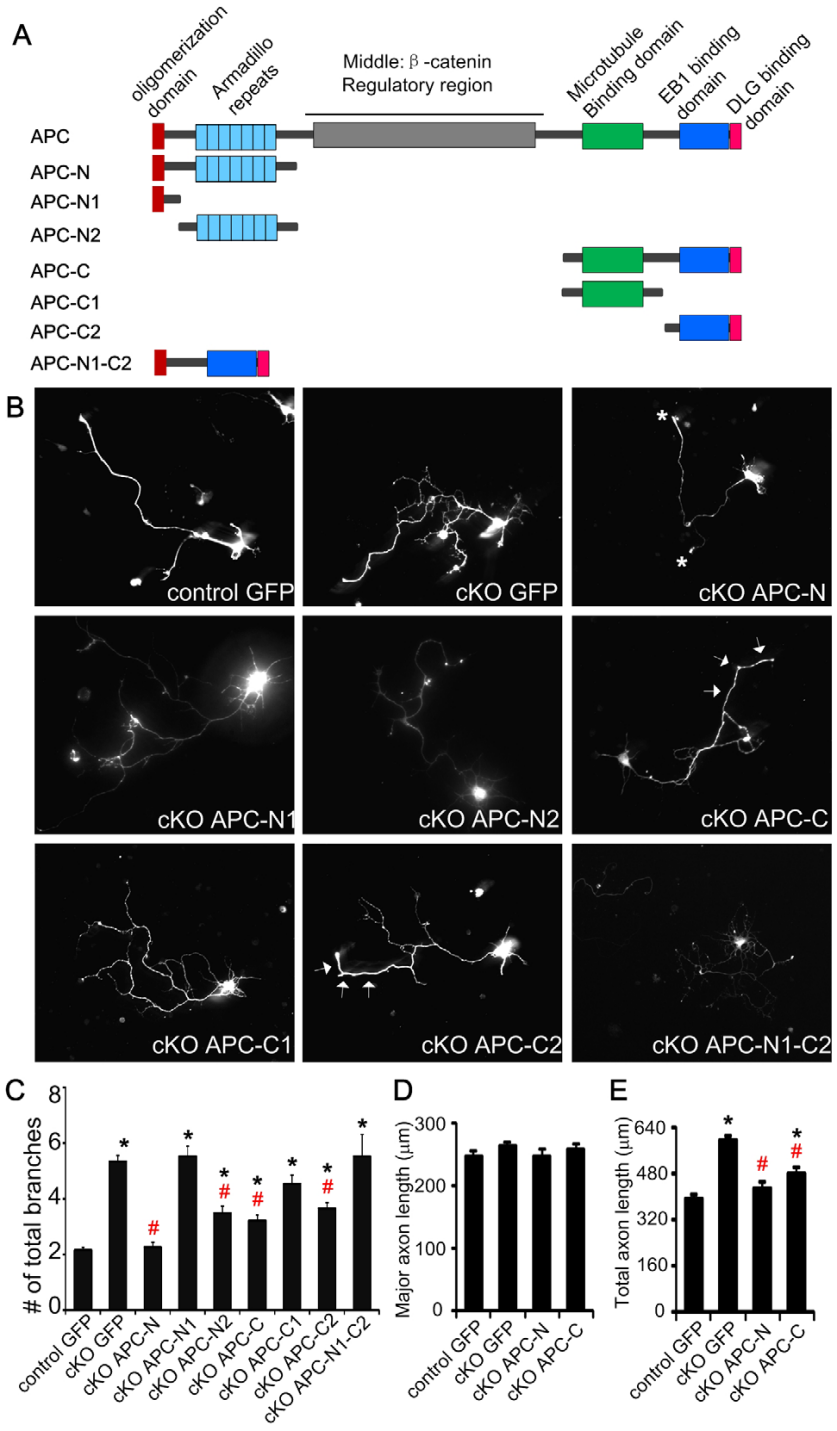
N-terminus of APC rescues branching phenotype in APC deleted neurons. (A) Schematic of full length APC and APC fragments used in this study. All truncation mutants were fused with GFP at the N-terminus. (B) Control cortical neurons expressing GFP alone and APC cKO cortical neurons expressing GFP alone or APC truncation mutants. Cortical neurons from E14.5–E15.5 embryos were dissociated, electroporated with GFP and the truncation mutants as indicated, and cultured for 4days *in vitro*. Representative images of axonal branching of neurons transfected with the indicated constructs are shown. Stars indicate the axon tip localization of APC-N. Arrows indicate the distal localization of APC-C and APC-C2. (C) Numbers of total axonal branches per neuron. (D) Average length of major axons and (E) total axon (including axon branches) length from neurons expressing GFP or APC truncation mutants as indicated. C, D, E: *: P<0.01, when compared with control neurons expressing GFP alone. #: P<0.01, when compared with APC deleted neurons expressing GFP alone. Note that APC-N fully rescued the excessive branching phenotype. n>140 neurons for each condition.

To investigate which specific domain of APC is responsible for regulating axonal branching and microtubule bundling, we expressed a series of GFP fused truncation mutants of APC (Fig. 5A) in APC deficient neurons and assessed their morphology after 4 days *in vitro* (Fig. 5B). The levels of GFP expression appeared similar for all of these constructs. Quantification of GFP fluorescence intensity of neurons expressing APC-N, APC-C, APC-C1 and APC-C2 in the same experiment is shown in Fig. S2.

In APC-deleted neurons (cKO GFP), total number of axonal branches was three times that of controls (Fig. 5B, C; * indicates significantly different from control). Remarkably, we found that expression of the N-terminal of APC (APC-N, containing both the oligomerization domain and the armadillo repeats) completely rescued the excessive branching caused by deletion of APC (Fig. 5B, C; # indicates significantly different from GFP expressing APC cKO neurons). In neurons expressing APC-N, the number of primary branches and total number of branches were similar to those of control neurons expressing GFP alone.

The N-terminus contains two distinct functional domains, the oligomerization domain and the armadillo repeats. To tease out which domain within the N-terminus of APC suppresses axon branching, we expressed the oligomerization domain (APC-N1) and the armadillo repeats (APC-N2) in APC-deficient neurons respectively. Expression of the oligomerization domain (APC-N1) in cortical neurons did not reduce excessive axon branching. Interestingly, expression of the armadillo repeats (APC-N2) only partially rescued the branching phenotype. Thus, neurons expressing APC-N2 had numbers of branches significantly different from both control GFP (*) and cKO GFP (#) transfected neurons. These results demonstrate that both the oligomerization domain and the armadillo repeats within the N-terminus of APC are important for fully suppressing excessive axonal branching.

Since the carboxyl (C) -terminal of APC contains the microtubule binding domain and the EB1 binding domain which are important for regulating microtubule dynamics, we initially hypothesized that expression of either the microtubule binding domain (APC-C1) or the EB1 binding domain (APC-C2) or both (APC-C) might fully rescue the branching phenotype of APC-deficient neurons. However, the C-terminal APC deletion mutants (APC-C, containing both microtubule binding domain and EB1 binding domain) only partially rescued the branching phenotype (Fig. 5B, C). Thus neurons expressing APC-C still elaborated twice as many branches as control GFP expressing neurons. Surprisingly expression of the microtubule binding domain (APC-C1) did not rescue excessive branching at all. Expression of the EB1 binding domain alone (APC-2) also only partially rescued the phenotype. We wondered whether fusing the oligomerization domain to EB1 might enhance EB1 effects, so we fused the oligomerization domain with the EB1 binding domain (APC-N1-C2) and examined the effect. However, we did not observe rescue of branching phenotype by APC-N1-C2.

Along with the branching effect, we also examined the localization of the APC truncations in the axons of APC deficient neurons. Interestingly, APC-N containing both the oligomerization domain and the armadillo repeats was accumulated at the axon tip (Fig 5B, stars). The armadillo repeats alone, APC-N2, was distributed evenly long the axon, with occasional accumulation at the tip. APC-C and APC-C2 showed a gradient distribution along the axon, with a strong localization at the distal part of the axon (arrows). All the other truncation mutants were distributed evenly along the axon. These results suggest that the N-terminus of APC can be targeted to the axon tip and is sufficient to suppress growth cone splitting.

APC-N has a dominant inhibitory effect in neurons with endogenous APC. Expression of APC-N in hippocampal and dorsal root ganglion neurons inhibits neuronal polarization and neurite outgrowth [14], [15]. We investigated whether the effects of APC-N in suppressing axon branching was caused by slowing down axon outgrowth. We measured the length of major axons and the total length of axons including branches (Fig. 5D and E). The average length of major axons from GFP expressing APC null neurons was similar to that of GFP expressing control neurons, while the total length was significantly increased in GFP expressing APC null neurons. Interestingly, expression of APC-N did not slow down axon outgrowth. The average length of major axons and the total length of axons were comparable to those GFP expressing control neurons. Our data suggest that the effect of APC-N in suppressing axon branching is not due to a general inhibition of axon outgrowth.

### N-APC rescues microtubule debundling

**Figure 6:**
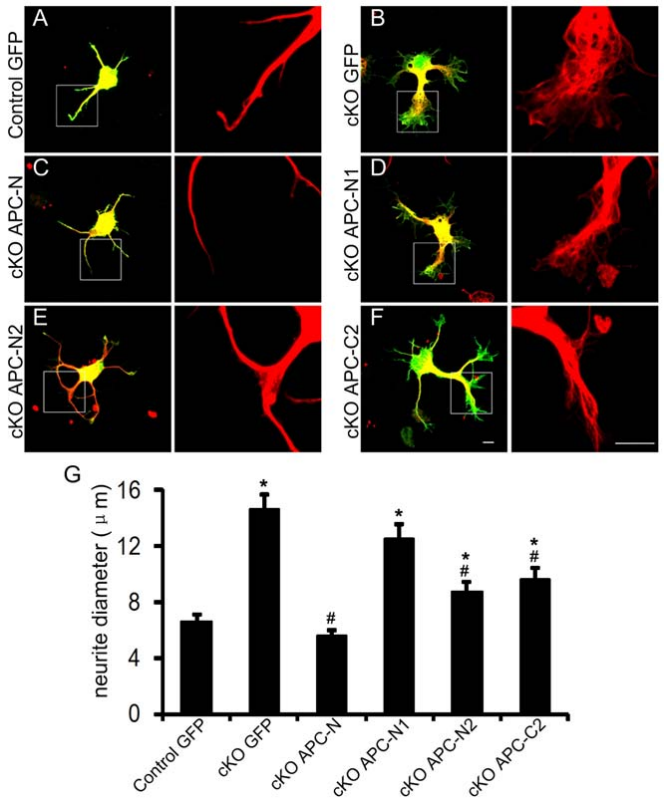
N-terminus of APC rescues microtubule debundling phenotype in APC deleted neurons. (A–F). Neurons were electroporated with GFP or APC truncation mutants after dissociation from E14.5–E15.5 embryo cortices. After 24 hours double label immunofluoresence was performed with antibodies against α-tubulin (red) and GFP (green). Right panels are the magnified views of neurites labeled with microtubule from left panels. (G) Neurite diameters measured from the thickest point. *: P<0.05, when compared with control neurons expressing GFP alone. #: P<0.05, when compared with APC deficient neurons expressing GFP alone. Note that expression of APC-N fully rescues the debundling phenotype. n = 30 neurons for each condition. Scale bars: 5 µm.

Since microtubules are highly debundled at the distal part of APC-deficient neurites at 1DIV, we next examined whether microtubule organization was rescued by any APC truncation mutants. To assess the microtubule debundling effect, we measured the neurite diameter at the thickest point of each neuron. The diameter of neurites from APC deleted neurons expressing GFP alone is more than two fold that of control neurons expressing GFP alone (Fig. 6A, B and G). Importantly, expression of the N-terminus of APC including both the oligomerization domain and the armadillo repeats (APC-N) completely rescued the microtubule debundling effects (Fig. 6C), and neurite diameter of APC-N expressing neurons was similar to that of control neurons expressing GFP alone (Fig. 6G). As with axon branching, APC-N1 alone did not rescue microtubule debundling (Fig. 6D, G) and expression of the armadillo repeats alone (APC-N2) only partially rescued. Expression of the EB1 binding domain (APC-C2) only partially rescued the microtubule debundling effect (Fig 6E, F). Taken together, our results suggest that the N-terminus of APC including the oligomerization domain and the armadillo repeats is both necessary and sufficient to rescue the disrupted microtubule organization and excessive branching of APC-deficient neurons.

## Discussion

By analyzing APC deficient neurons in dissociated cortical cultures, we have provided new insights into mechanisms of APC regulation of axonal development. First, we found that deletion of APC leads to excessive axon branching and many of the exuberant branches arise from growth cone splitting. Second, APC is essential for microtubule and actin organization within the neurites and growth cones of early developing mouse cortical neurons. Finally, we have demonstrated that expression of the N-terminus can completely rescue morphological abnormalities associated with APC deletion in cortical neurons. Our data are consistent with the surprising notion that the amino terminus of APC is important for axonal morphogenesis.

### Cytoskeletal abnormalities in APC deficient neurons

Our study revealed a variety of morphological abnormalities in APC deleted cortical neurons. However, we found that initial polarization occurs normally. Thus, specification of the axons and establishment of axonal and somatodendritic domains proceeded in the absence of APC. These results are surprising in view of previous studies using dominant negative approaches *in vitro* in both non-neuronal cells and neurons that have suggested an important role for APC in regulating cell polarity [2], [12], [14], [32], [33], [34]. One explanation for this discrepancy is that dominant negative approaches may have unexpected consequences related to sequestering binding partners and disrupting their normal functions. In fact, a recent study in Drosophila also demonstrated that APC is dispensable for establishing neuronal polarity [35].

A major abnormality we observed from APC deficient cortical neurons is the disruption of growth cone cytoskeleton organization. Microtubules are misorientated, highly de-fasciculated and extend randomly to the leading edge without restraint from the F-actin barrier. The microtubule misorientation appears to be quite general as a previous study in mouse DRG neurons, reported that reducing endogenous APC protein by shRNA induces microtubule loop formation in the growth cone together with increased growth cone size [6]. Here, we further demonstrated that APC plays a key role in the maintenance of growth cone organization during axon extension.

In association with the microtubule abnormalities, we observed an enlargement of the growth cone size together with disrupted actin organization as evidenced by aberrant microtubule extension to the growth cone tip. A plausible explanation for our results would be that F-actin disruption in APC deficient growth cones prevents proper regulation of microtubule extension. Indeed, the interaction between microtubules and F-actin is known to be important during axon guidance and growth cone steering [26]. Interestingly, previous studies have demonstrated that splaying microtubules and increased filopodia coincide with the development of axon branches from the growth cone [36], [37]. In fact, our time lapse imaging results showed that these enlarged APC deficient growth cones split apart frequently, producing multiple branches at the time of splitting.

### The N-terminus of APC is necessary and sufficient to rescue neuronal morphological abnormalities

Specification of the domain or domains involved should provide important insights into how APC regulation of axon morphology is mediated. In our study, we observed an increase in β-catenin protein levels in APC deficient neurons as expected. However, by expressing a non-degradable β-catenin mutant in control neurons and a β-catenin inhibitor in APC deficient neurons, we demonstrate that excessive branching in APC null axons is not caused by stabilized β-catenin. Thus perhaps surprisingly, our results suggest that regulation of β-catenin by APC does not appear to be involved in axon morphogenesis. Previous studies have suggested that stabilized β-catenin inhibits neurite outgrowth from PC12 cells and mouse retinal cells [24], [38] while another study suggests that β-catenin stabilization increases dendritic arborization in hippocampal neurons [30]. Our findings are consistent with this latter report.

While mutations of APC found in FAP patients disrupt or delete a large part of the protein, the amino-terminus including the oligomerization domain and the armadillo repeats of APC are typically retained and protected from mutations. APC deleted neurons provided the ideal assay system to assess effects of the N-terminus on the neuronal cytoskeleton. Interestingly, we found that expression of APC-N completely rescues growth cone cytoskeletal abnormalities and the branching phenotype of APC deficient axons. N-terminus regulation may be due to the armadillo repeats in this region. The armadillo repeats bind to several proteins that are involved in regulation of the cytoskeleton including ASEF and IQGAP and regulate the activities of these molecules [7]. Interaction of the armadillo repeats with ASEFs and IQGAP are involved in the formation of membrane ruffles [7], [9], [10], [39], [40], [41], [42]. These effects may explain the striking increase in filopodia and disruption of the F-actin barrier that we have observed in APC-deficient growth cones. Further, interaction of IQGAP with Clip-170 recruits microtubule plus ends to the actin meshwork through association with Rac1/Cdc42 [10]. Finally, the armadillo repeats also interact with kinesin associated protein (KAP3) which is a subunit of kinesin-2 [8]. Recent evidence in Drosophila suggests that interaction of APC with kinesin-2 is involved in the microtubule directional growth [43]. Loss of this mechanism might explain the striking disruption of microtubule orientation in the growth cone due to APC deletion.

Interestingly, expression of the armadillo repeats alone is not sufficient to rescue either the branching effect or the cytoskeletal abnormalities observed in APC-deficient neurons. The oligomerization domain is also required. We propose that dimerization of the armadillo repeats may promote the interaction of APC with different components of the actin and microtubule regulating machinery and promotes microtubule-F-actin coupling during axon extension.

Previously, the N-terminus of APC was shown to suppress axon extension in DRG neurons [15] and inhibit neuronal polarization in developing hippocampal neurons [14]. In our study of cortical neurons neither initial extension of the axon nor total axonal length were affected. Differences in interpretation from these previous investigations may relate to the fact that endogenous APC was absent from the neurons that we studied. Indeed, the N-terminus of APC is thought to act as a dominant-negative mutant when endogenous APC is present and further may have off target effects including suppression of function of a closely related family member, APC2 [44].

We initially hypothesized that the microtubule binding domain and EB1 binding domain at the C-terminus of APC would rescue the branching phenotype. However, the N-terminal fragment that completely rescues neuronal morphology does not contain these regions. Further, reintroduction of the C-terminus only partially suppresses axon branching, and the microtubule debundling observed at the early stages was not rescued either. Moreover, the microtubule binding domain has no effect on axon branching.

### Conclusion

In conclusion, our data demonstrate that the N-terminus of APC has important functions in the regulation of neuronal cytoskeleton. The associations of the N-terminus with KAP3, ASEFs, IQGAP and Clip-170 may explain why reintroducing N-terminus is sufficient to correct APC deletion induced cytoskeleton reorganization. Importantly, mice homozygous for an APC truncation mutation lacking the C-terminus microtubule and EB1 binding domains are viable which further bolsters our conclusion about the importance of the N-terminus to neuronal development [45].

## Supporting Information

Figure S1 **Quantification of APC staining at the growth cone.** Fluoresence intensity is expressed as a ratio of APC staining normalized to α-tubulin staining. 11 control growth cones and 25 growth cones from APC^lox/lox^Nestin-Cre^+^ mice were assessed. ***: P<0.001 by student t test.
(TIF)

Figure S2 **Fluorescent intensity of APC-N, APC-C, APC-C1 and APC-C2 expression in APC deficient neurons.** Data are shown from a representative experiment. Histograms represent integrative GFP fluorescent intensity of neurons expressing each fragment measure by Metamorph software. Note that no significant differences were observed among these deletion mutants.
(TIF)
