# Supplementary figures and images for: Adenomatous Polyposis Coli Regulates Axon Arborization and Cytoskeleton Organization via Its N-Terminus
Source: PLoS One. 2011 Sep 6;6(9):e24335. doi: 10.1371/journal.pone.0024335 (PMC3167844; doi:10.1371/journal.pone.0024335)

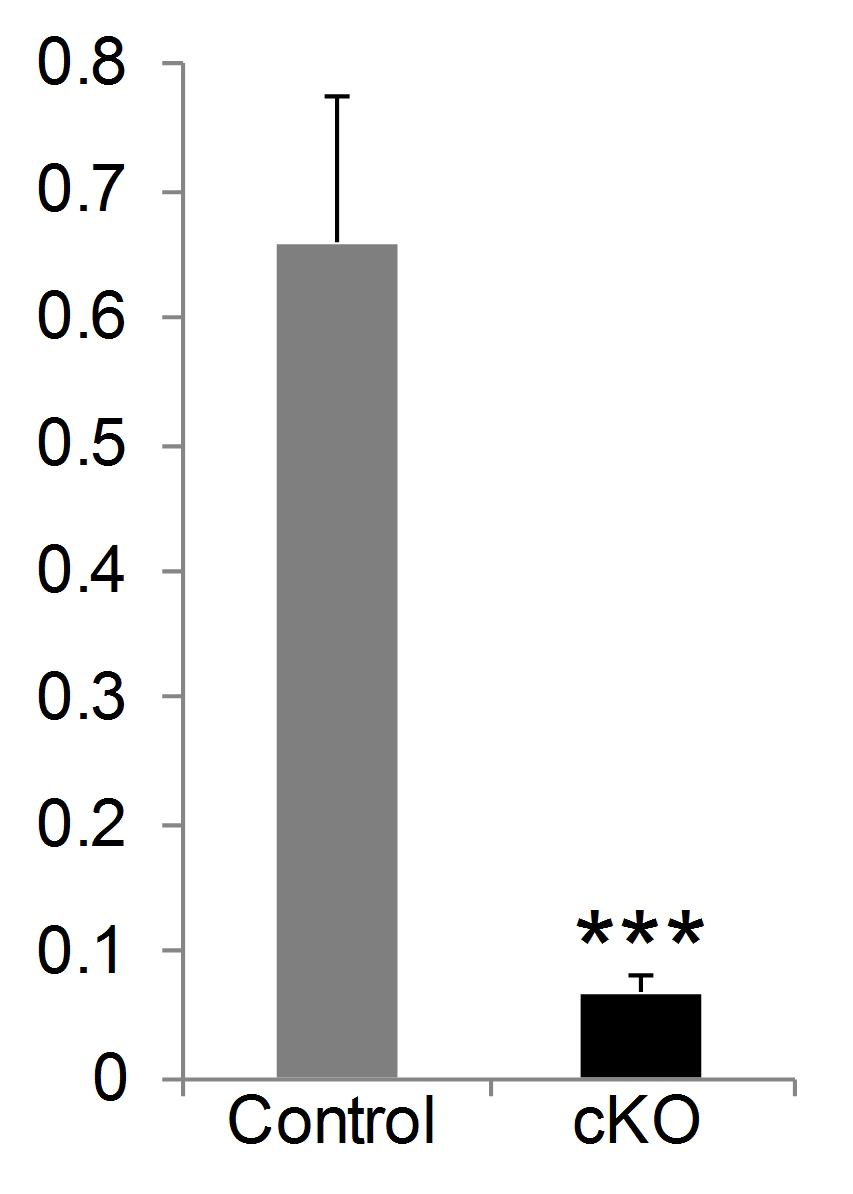

Supplement: Figure S1 — Quantification of APC staining at the growth cone. Fluoresence intensity is expressed as a ratio of APC staining normalized to α-tubulin staining. 11 control growth cones and 25 growth cones from APClox/loxNestin-Cre+ mice were assessed. ***: P<0.001 by student t test. (TIF) [file pone.0024335.s001.tif]

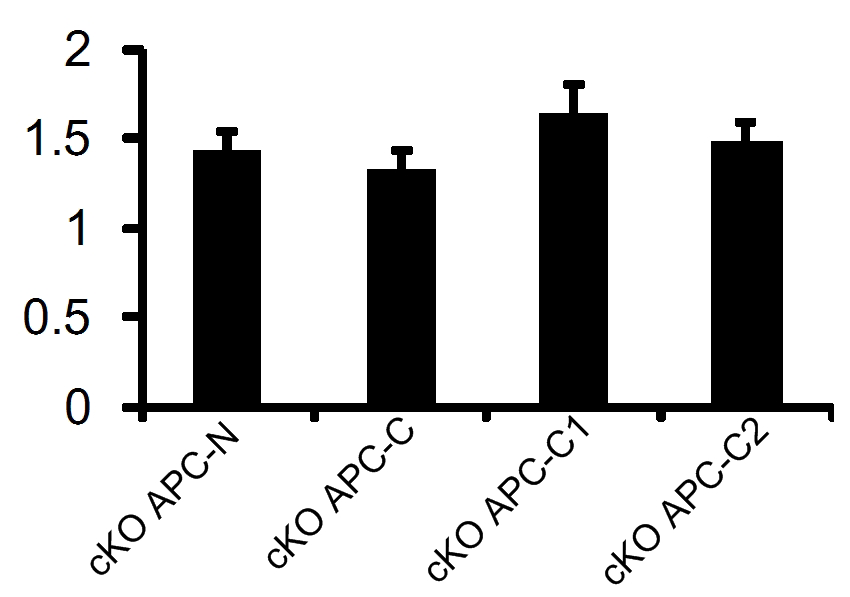

Supplement: Figure S2 — Fluorescent intensity of APC-N, APC-C, APC-C1 and APC-C2 expression in APC deficient neurons. Data are shown from a representative experiment. Histograms represent integrative GFP fluorescent intensity of neurons expressing each fragment measure by Metamorph software. Note that no significant differences were observed among these deletion mutants. (TIF) [file pone.0024335.s002.tif]
